# Supplementary material for: Interfacial self-healing polymer electrolytes for long-cycle solid-state lithium-sulfur batteries
Source: Nat Commun. 2024 Jan 8;15:351. doi: 10.1038/s41467-023-43467-w (PMC10774406; doi:10.1038/s41467-023-43467-w)
Supplement: Supplementary file 1 — Supplementary Information [file 41467_2023_43467_MOESM1_ESM.pdf]

Supplementary Information

Interfacial self-healing polymer electrolytes for long-cycle solid-state lithium-sulfur batteries

Fei Pei,[1] Lin Wu,[1] Yi Zhang,[1] Yaqi Liao,[1] Qi Kang,[1] Yan Han,[1] Huangwei Zhang,[1] Yue Shen,[1] Henghui Xu,*[,1] Zhen Li*[,1] & Yunhui Huang*[,1]

[1]State Key Laboratory of Materials Processing and Die & Mould Technology
School of Materials Science and Engineering
Huazhong University of Science and Technology
Wuhan 430074, China

*Corresponding authors

E-mail: xuhh@hust.edu.cn; li_zhen@hust.edu.cn; huangyh@hust.edu.cn

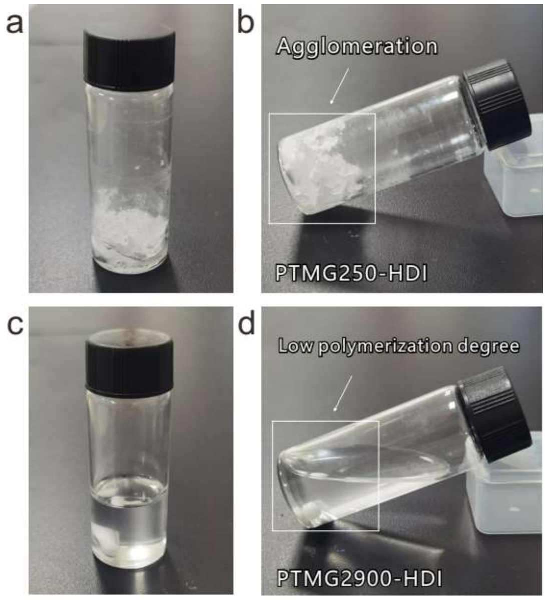

Supplementary Fig. 1 Effect of molecular weight (PTMG) on polymerization process. Digital photographs of the PTMG-HDI solutions with the (a, b) short-chain PTMG250 and (c, d) long-chain PTMG2900.

The molecular weight of the monomer (PTMG) had a significant effect on the polymerization process. When controlling for the same molar ratio (1:1.2) of monomer PTMG and HDI, the short-chain PTMG250 (Mw=250 g mol^{-1}) could react violently with HDI at low temperature, the reaction rate was uncontrollable and the obvious agglomeration occurred quickly. As another controlled experiment, the reaction rate of PTMG2900 (Mw=2900 g mol^{-1}) was quite slow even under the condition of higher temperature and extended reaction time. The final viscosity of the obtained polymer solution was obviously low, probably due to the poor reactivity of the terminal functional group of the long-chain PTMG2900 when compared with PTMG250. More seriously, the solution of polymerized PTMG2900 was further casted on polytetrafluoroethylene plate, the resulting dried polymer was too sticky to peel off. Therefore, the selection of monomers with appropriate molecular weight is very important for polymerization.

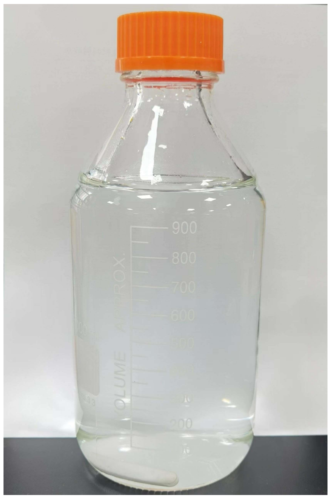

Supplementary Fig. 2 Digital photographs of the PTMG-HDI-BHDS solution (1000 mL) prepared by magnifying production.

The reaction conditions of stepwise polymerization are mild, uniform and controllable, very easy to scale up to the kilogram level, as shown in the Supplementary Fig. 2, we have successfully prepared PTMG-HDI-BHDS with a solid content of 100 g in a closed glass bottle. The solid content is 10 wt%.

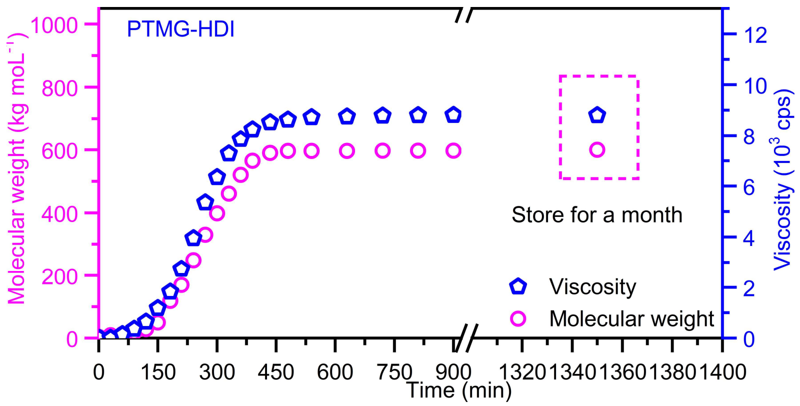

Supplementary Fig. 3 Variation processes of molecular weight (red point) and viscosity (blue point) during polymerization.

The molecular weight-viscosity data of PTMG-HDI was shown in the Supplementary Fig. 3, the viscosity increases synchronously with the during the reaction time for 8 h at 40 ^oC, the molecular weight (Mn) and viscosity (η) were increased to 5.97 x 10^5 g mol^{-1} and 0.88 x 10^4 cps, respectively, significantly lower than that of PTMG-HDI-BHDS. Polyurethane has excellent thermal and chemical stability, so it can be stored for a long time, for example, after one month, the viscosity and molecular weight of the synthesized polyurethane remain constant.

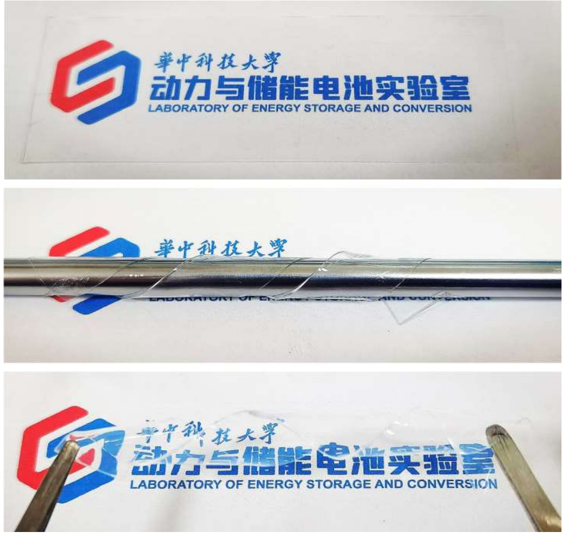

Supplementary Fig. 4 Digital photographs of the PTMG-HDI-BHDS films in various states.

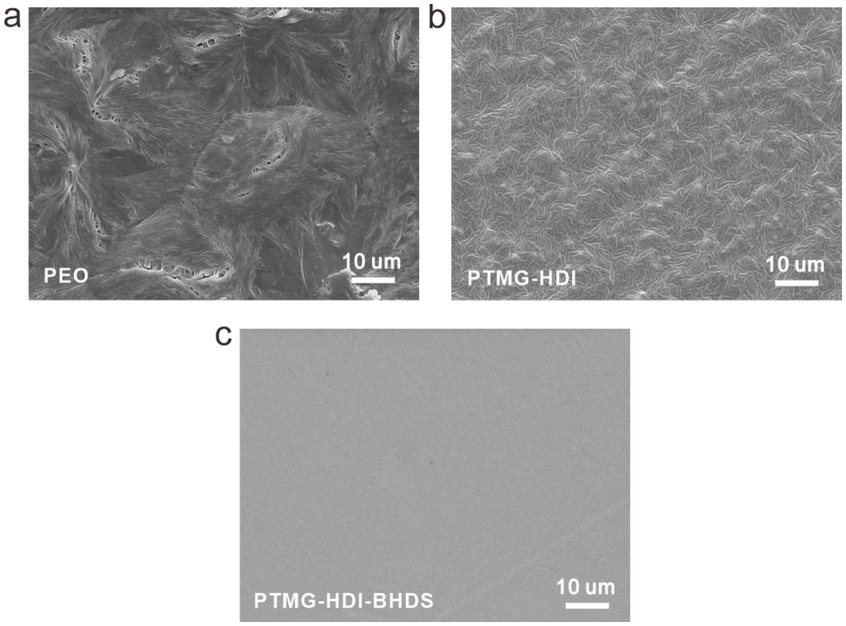

Supplementary Fig. 5 SEM images of the polymers. Top-view SEM images of (a) PEO, (b) PTMG-HDI and (c) PTMG-HDI-BHDS films.

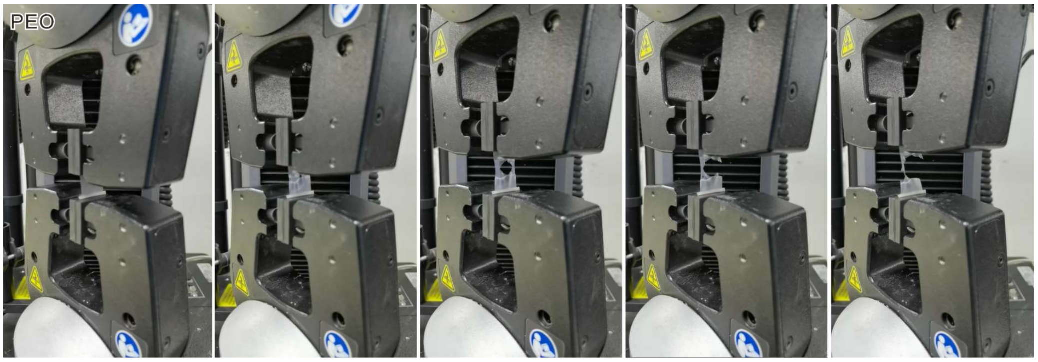

Supplementary Fig. 6 Photographs of the stress-strain measurement of PEO film.

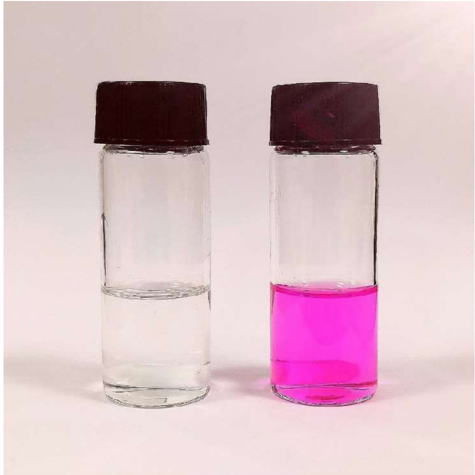

Supplementary Fig. 7 Photographs of before and after rhodamine B was added in the colorless PTMG-HDI-BHDS gel solution.

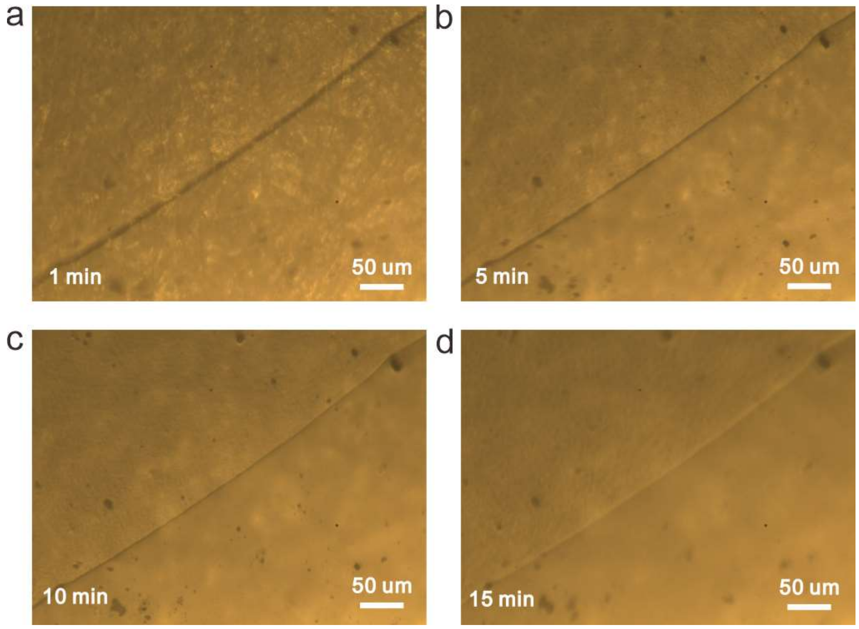

Supplementary Fig. 8 Optical microscopy images of the scratch on the PTMG-HDI-BHDS film before and after healing for 15 min at 30 ^{o}C. (a) 1 min, (b) 5 min, (c) 10 min, (d) 15 min. The optical microscope images showed that the scratches had almost disappeared after healing.

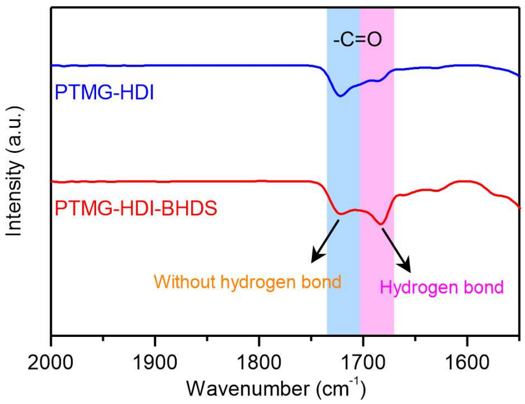

Supplementary Fig. 9 FTIR spectra of PTMG-HDI and PTMG-HDI-BHDS films.

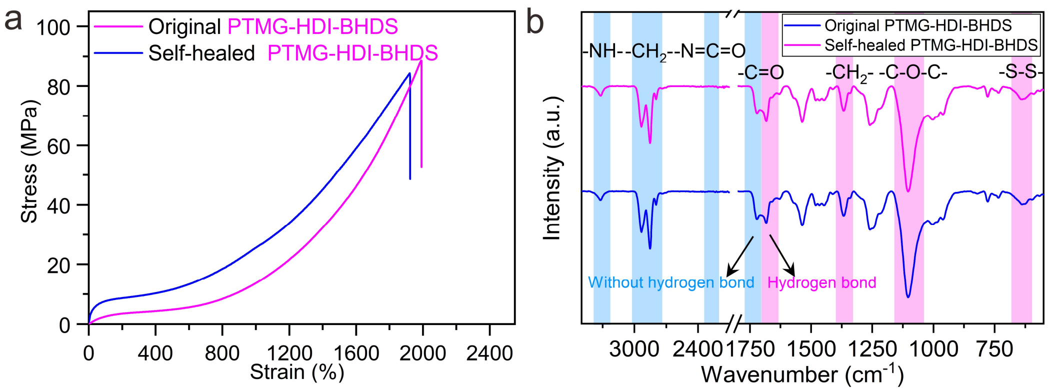

Supplementary Fig. 10 Comparison of tensile properties and FT-IR before and after self-healing. (a) Stress-strain measurements and (b) FT-IR analysis of the original and self-healed PTMG-HDI-BHDS.

The mechanical properties of the self-healed PTMG-HDI-BHDS film in Fig. 2d were further investigated to evaluate the self-healing ability. The break strength and ultimate elongation of the self-healed PTMG-HDI-BHDS were 84.3 MPa and 1920%, respectively, which were very close to the original value (Fig. 2d and Supplementary Fig. 10a), indicating that the self-healed fracture surface is well integrated in the presence of abundant hydrogen bonds and disulfide bonds, and can be restored to the original mechanical strength. The resulting characteristic functional groups of the pristine and self-healed of the SPEs were compared by ATR-FTIR (Fig. 2g and Supplementary Fig. 10b). It can be found that the characteristic peaks of all functional groups (e.g., -S-S-, -NH-COO-) are well coincident.

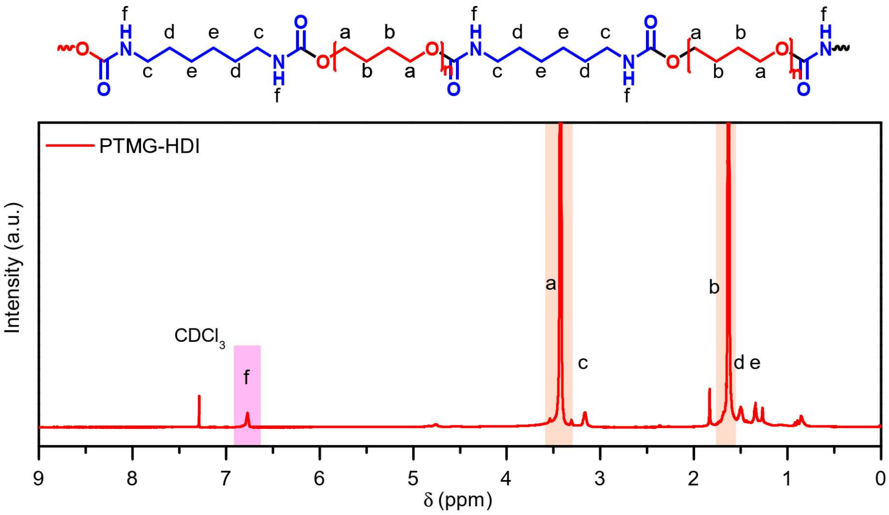

Supplementary Fig. 11 ^{1}H NMR spectrum of the synthesized PTMG-HDI in $CDCl_3$ (500 MHz).

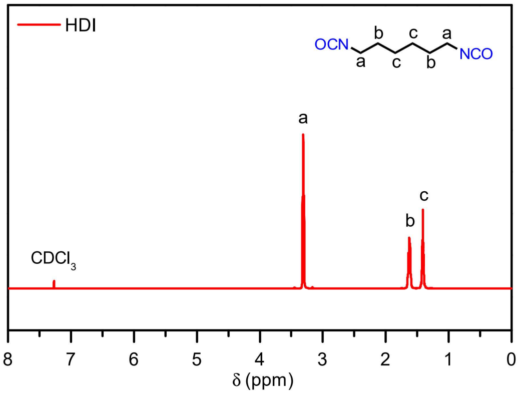

Supplementary Fig. 12 ^{1}H NMR spectrum of the HDI in $CDCl_3$ (500 MHz).

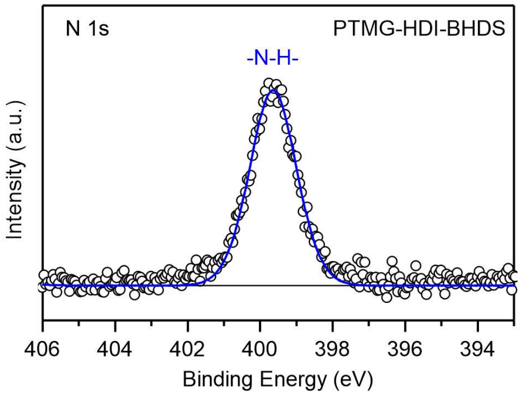

Supplementary Fig. 13 N *1s* XPS spectrum of PTMG-HDI-BHDS film.

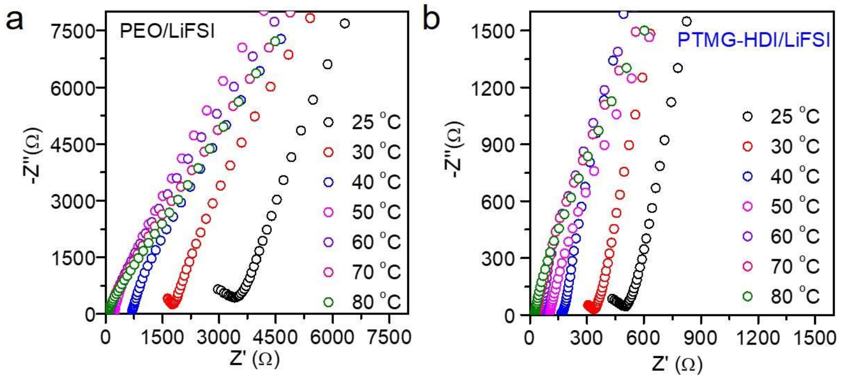

Supplementary Fig. 14 Electrolyte impedances under different temperatures. EIS measurements of the (a) PEO/LiFSI and (b) PTMG-HDI/LiFSI at different temperatures.

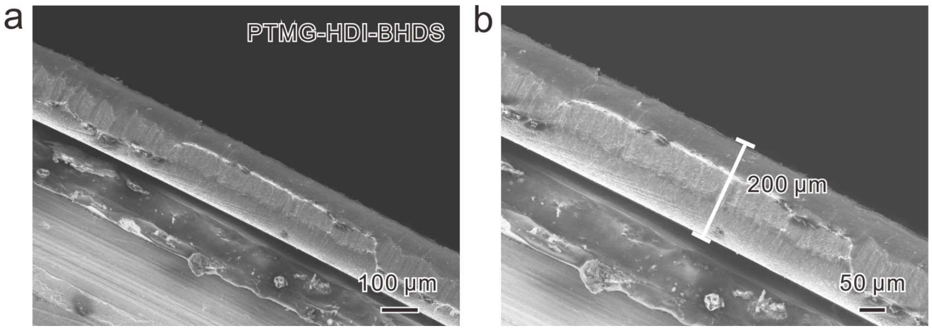

Supplementary Fig. 15 Morphology of the cross section of the electrolyte film. (a) Cross-sectional SEM image of PTMG-HDI-BHDS/LiFSI film. (b) Cross-sectional SEM image of PTMG-HDI-BHDS/LiFSI film.

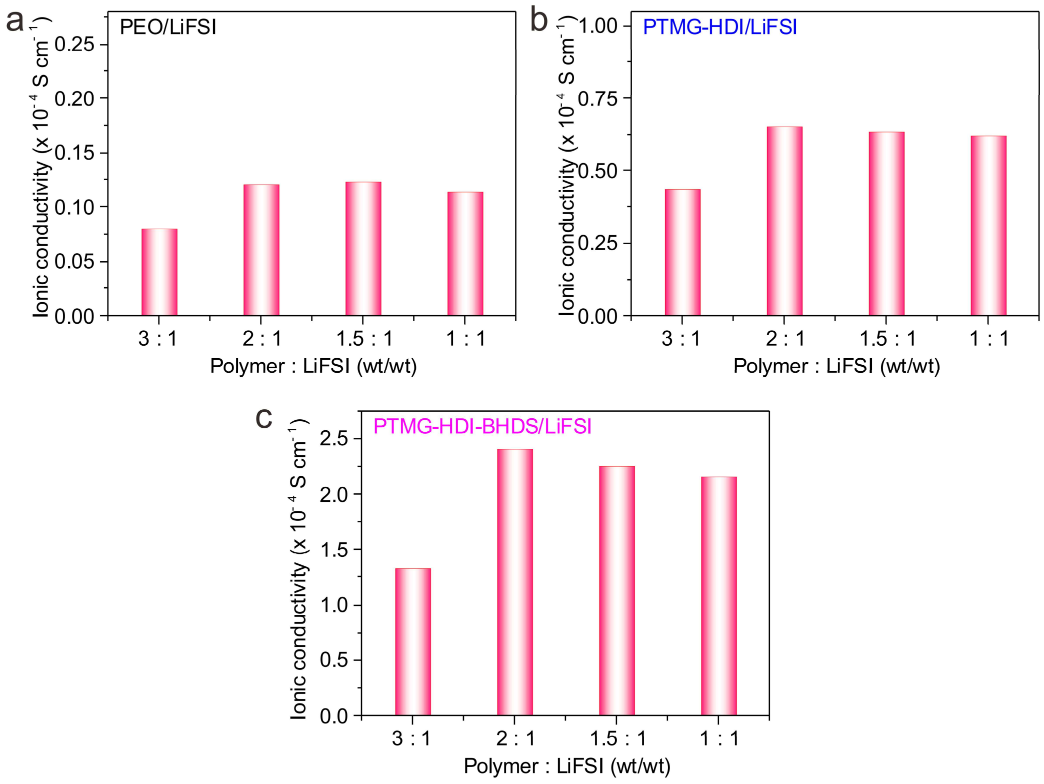

Supplementary Fig. 16 Effect of LiFSI content on ionic conductivity. The effect of lithium salt (LiFSI) content on ionic conductivity of (a) PEO/LiFSI, (b) PTMG-HDI/LiFSI and (c) PTMG-HDI/LiFSI.

The effect of lithium salt (LiFSI) content on ionic conductivity was provided in the revised manuscript. The ratios between the polymer and LiFSI were set to 3:1, 2:1, 1.5:1 and 1:1, we found that under the condition of proper concentration of LiFSI (mass ratio of 2:1), the PTMG-HDI/LiFSI and PTMG-HDI-BHDS/LiFSI were overall in the highest range of ionic conductivity. For the PEO/LiFSI, when the ratio was 1.5:1, the conductivity reached the highest value (1.23×10^{-5} S cm^{-1}), but was not much different from the conductivity of the ratio of 2:1 (1.20×10^{-5} S cm^{-1}). High content of LiFSI did not significantly improve the conductivity, but seriously reduced the mechanical strength of the SPEs

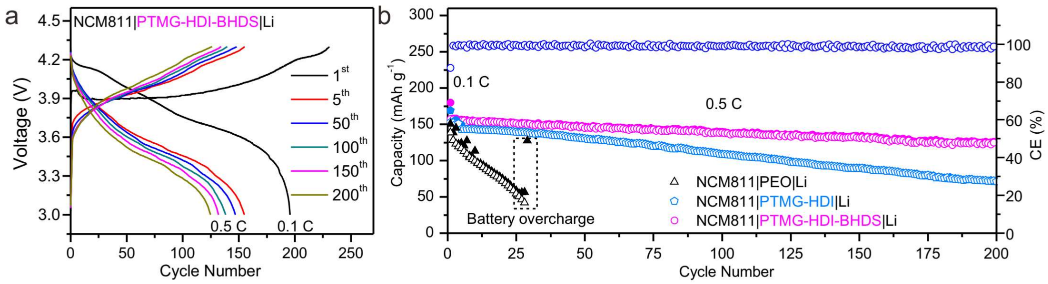

Supplementary Fig. 17 Long-term cycling performances of NCM811|SPEs|Li cells. (a) Voltage profiles of the NCM811|PTMG-HDI-BHDS|Li cell at 0.5 C. (b) Long-term cycling performance of NCM811|SPEs|Li cell at 0.5 C.

High-voltage Li-metal batteries (HVLMBs) coupling with solid-state polymer electrolytes (SPEs) have been regarded as a promising strategy to guarantee the high energy density and safety. The target SPE can effectively suppress the decomposition at high oxidation potential (5.1 V), showing advantages in $LiNi_{0.8}Co_{0.1}Mn_{0.1}O_2$ (NCM811) battery systems. As a result, the assembled NCM811|PTMG-HDI-BHDS|Li cell delivering a high discharge capacity of 157.5 mAh g^{-1} with a capacity retention of 80.0% over 200 cycles, effectively suppressing the microstructural degradation and side reactions of NCM811. The cathode slurry was obtained by mixing the NCM811, CB, SPEs and PVDF with a weight ratio of 80 : 10 : 5 : 5 in DMAc. The as-obtained slurry was then coated onto carbon-coated Al foil, dried at 65 °C in vacuum with a mass loading of 3 mg cm^{-2}.

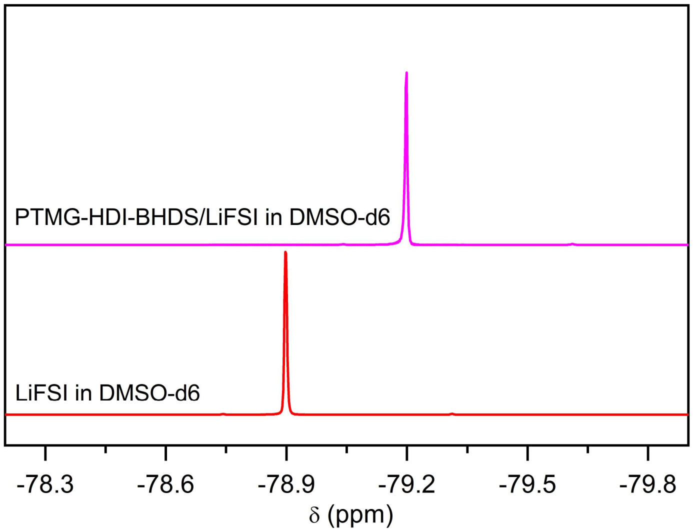

Supplementary Fig. 18 The ^{19}F NMR spectra of LiFSI in DMSO-*d*6 without or with presence of PTMG-HDI-BHDS.

The formation of hydrogen bonds between H atom and F in anions of fluoride salts (LiTFSI and LiFSI) has been demonstrated in the literature by NMR[1]. Considering that the anions contain a lot of F atoms and the H···F bonds are the strongest hydrogen bonds. The abundant urethane groups in PTMG-HDI-BHDS are able to provide a rich hydrogen bond network with FSI^- anions, significantly blocking the free movement of FSI^- to a certain extent. The formation of -NH-···F hydrogen bonds between urethane groups and FSI^- can be further confirmed by the upfield displacement of the chemical shift of FSI^- in the ^{19}F spectra (Supplementary Fig. 18). The abundant ether oxygen ($EO-Li^+$) and carbonyl oxygen functional groups in the structure promoted the dissociation of LiFSI and significantly promoted the free movement of Li^+. When the above two effects acted at the same time, the number of Li^+ transference number was significantly increased.

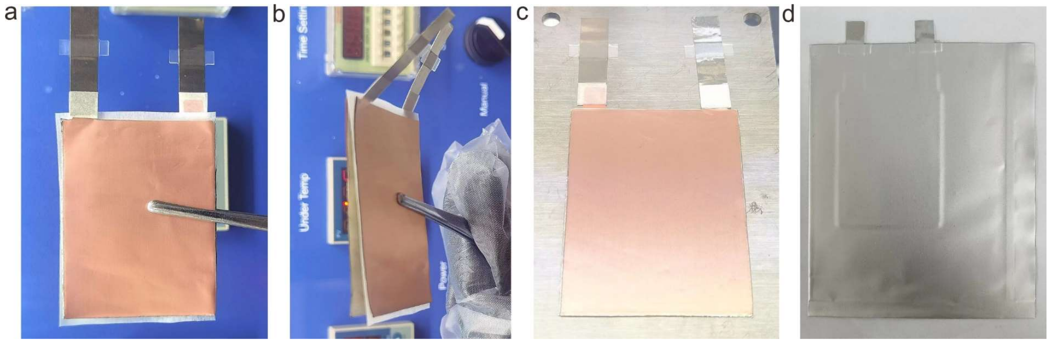

Supplementary Fig. 19 Digital photographs of the conventional (a, b) laminated Li|PEO|Li pouch cell and (c, d) integrated Li|PTMG-HDI-BHDS|Li pouch cell.

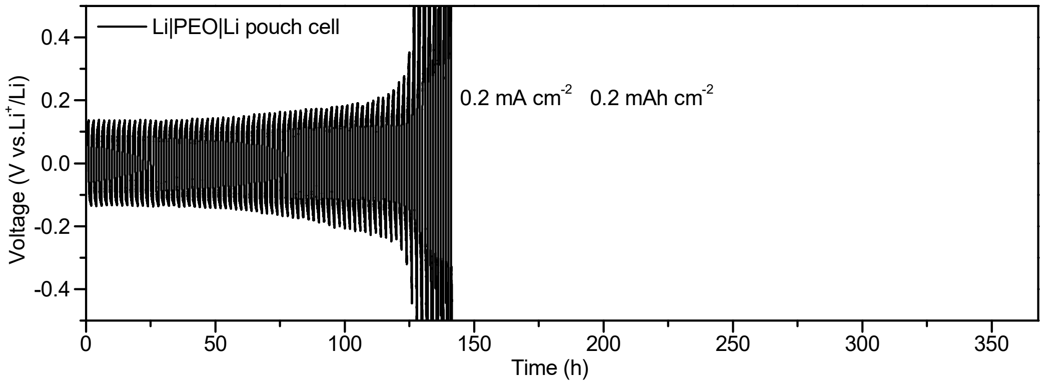

Supplementary Fig. 20 Galvanostatic cycling of Li|PEO|Li pouch cells at the current density of 0.2 mA cm^{-2} for 140 h.

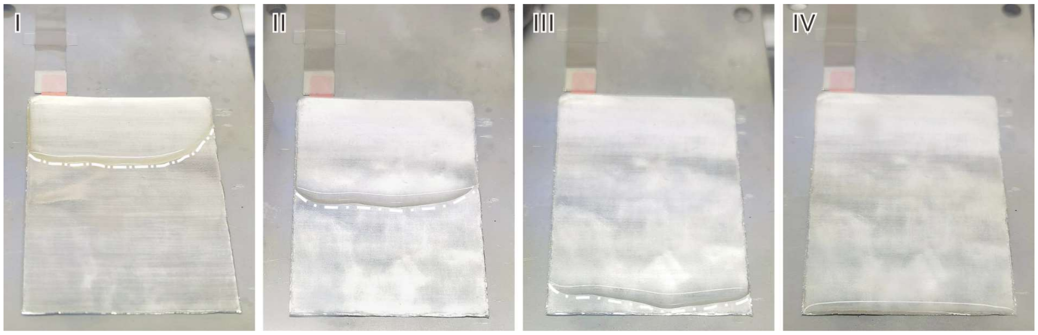

Supplementary Fig. 21 The SPEs solution were introduced on the Li anodes through casting infiltration. The integrated Li@SPE were formed by the subsequent solvent evaporation.

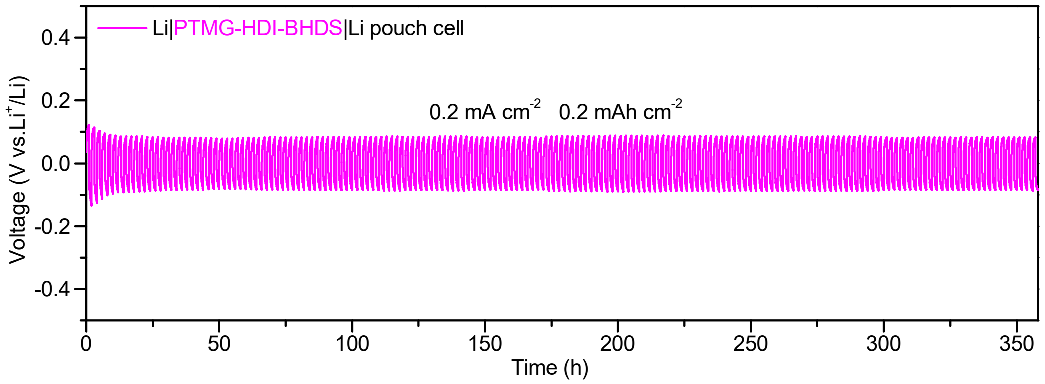

Supplementary Fig. 22 Galvanostatic cycling of Li|PTMG-HDI-BHDS|Li pouch cells at the current density of 0.2 mA cm^{-2}.

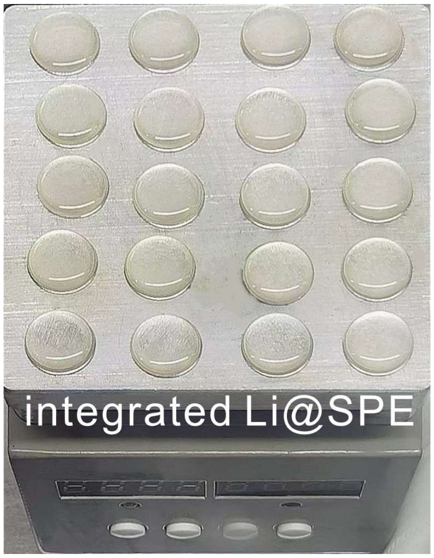

Supplementary Fig. 23 Digital photograph of the integrated Li@SPE before drying.

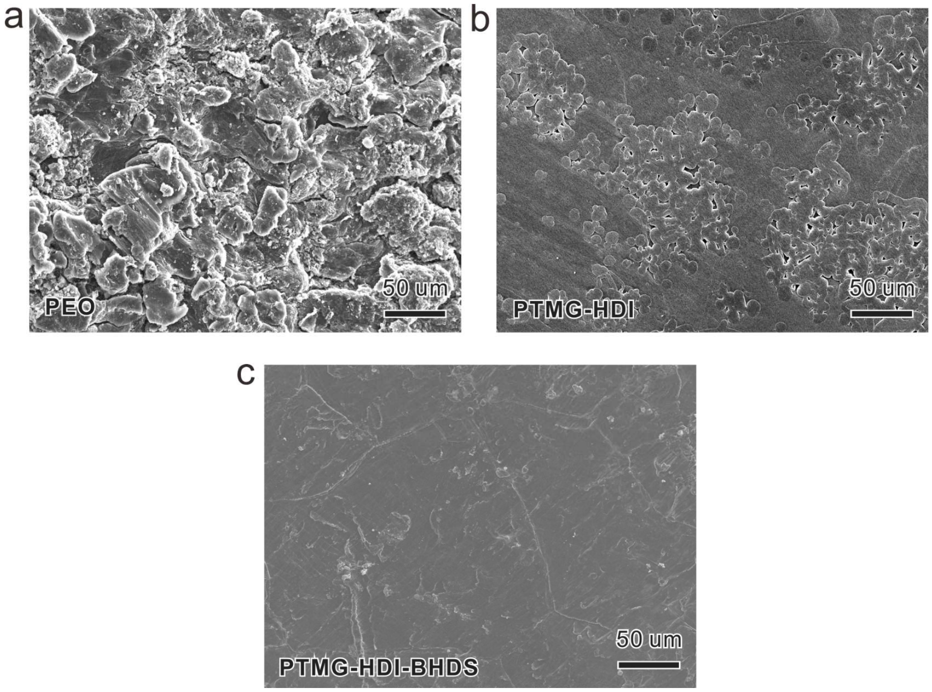

Supplementary Fig. 24 Morphologies of the cycled Li anodes. Top view SEM images of the cycled Li anodes detached from (a) Li|PEO|Li, (b) Li|PTMG-HDI|Li and (c) Li|PTMG-HDI-BHDS|Li cells.

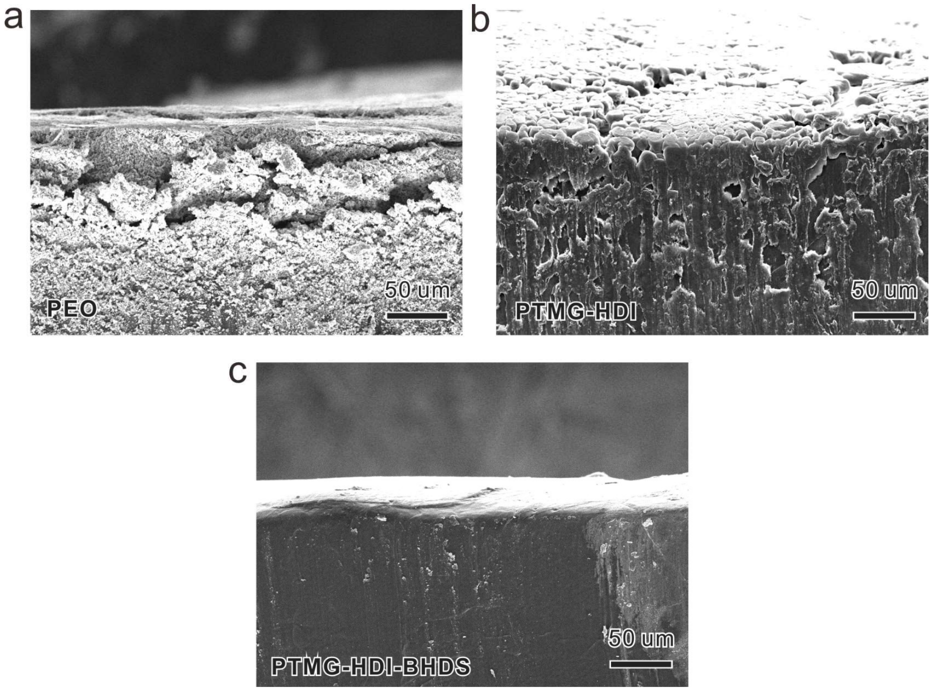

Supplementary Fig. 25 Morphologies of the cycled Li anodes. Cross-sectional SEM images of the cycled Li anodes detached from (a) Li|PEO|Li, (b) Li|PTMG-HDI|Li and (c) Li|PTMG-HDI-BHDS|Li cells.

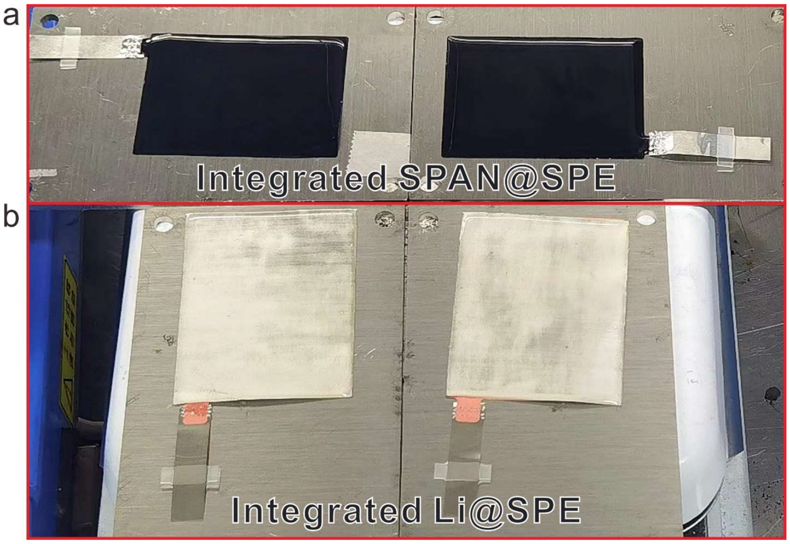

Supplementary Fig. 26 Digital photographs of the integrated electrodes. (a) Photographs of SPAN@SPE. (b) Photographs of Li@SPE.

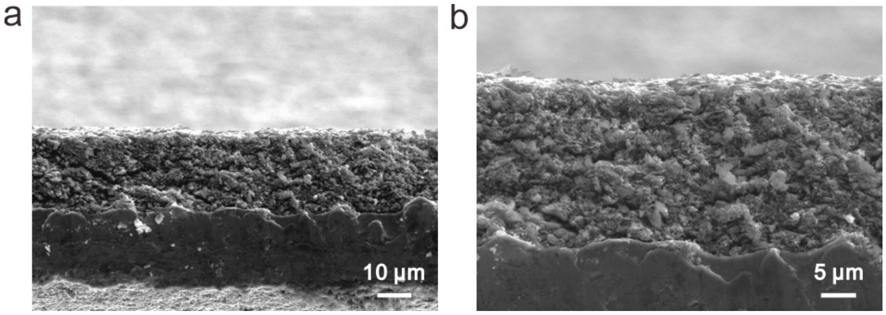

Supplementary Fig. 27 Morphologies of SPAN cathodes. (a) The cross-sectional SEM image of SPAN@SPE cathode. (b) The enlarged cross-sectional SEM image of SPAN@SPE cathode.

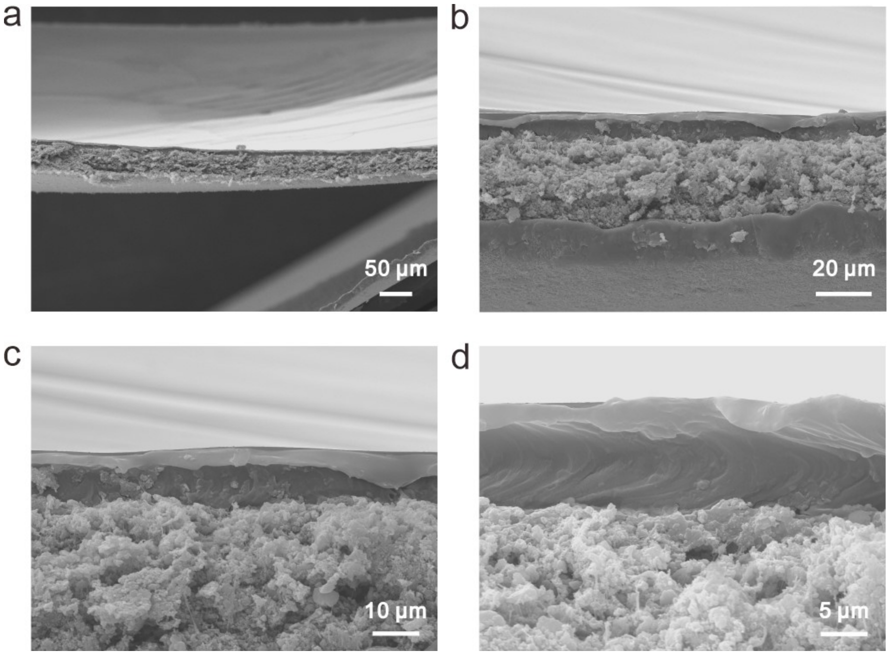

Supplementary Fig. 28 Morphologies of integrated SPAN@SPE cathodes. (a, b) The cross-sectional SEM images of SPAN@SPE cathode. (c, d) The enlarged cross-sectional SEM images of SPAN@SPE cathode.

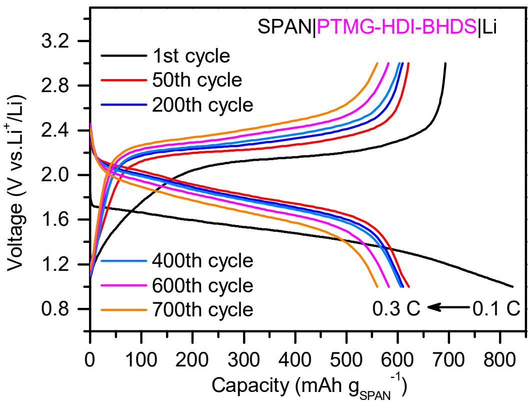

Supplementary Fig. 29 Charge/discharge curves of the SPAN|PTMG-HDI-BHDS|Li cell at 0.3 C.

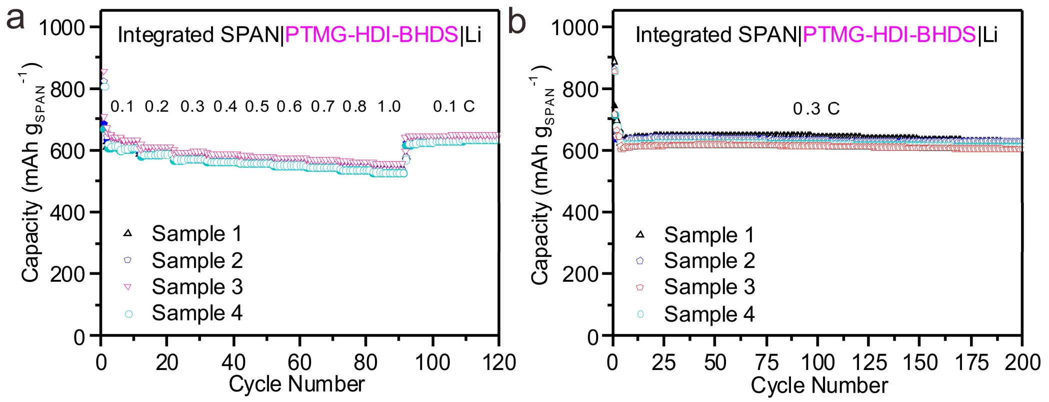

Supplementary Fig. 30 Reproducibility of the cell testing. (a) Rate and (b) cycling performance of different SPAN|PTMG-HDI-BHDS|Li cells with a mass loading of 2.0~2.2 mg cm^{-2} tested with the same experimental conditions.

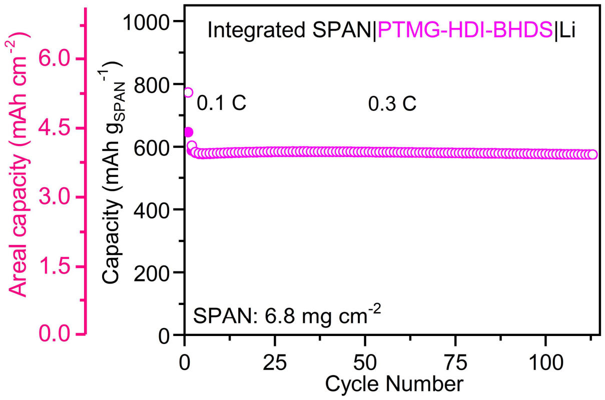

Supplementary Fig. 31 Cycling performance of the SPAN|PTMG-HDI-BHDS|Li at 0.3 C with a high loading of 6.8 mg cm^{-2}.

We demonstrated that PTMG-HDI-BHDS/LiFSI was capable of supporting higher cathode loading, which was important to meet the requirement for the state-of-the-art lithium batteries. The designed SPE acted as both a Li^+ conductor and binder (experimental section), providing Li^+ pathways in high loading thick SPAN cathode (6.8 mg cm^{-2} with a thickness of ~80 μm) (Supplementary Fig. 35). The SPAN|PTMG-HDI-BHDS|Li cell delivered a high discharge capacity of 647 mAh g^{-1} with a high areal capacity of 4.4 mAh cm^{-2} and kept stable over 110 cycles (Supplementary Fig. 31). This work provided a promising strategy for the design of high-energy-density solid-state Li-S batteries.

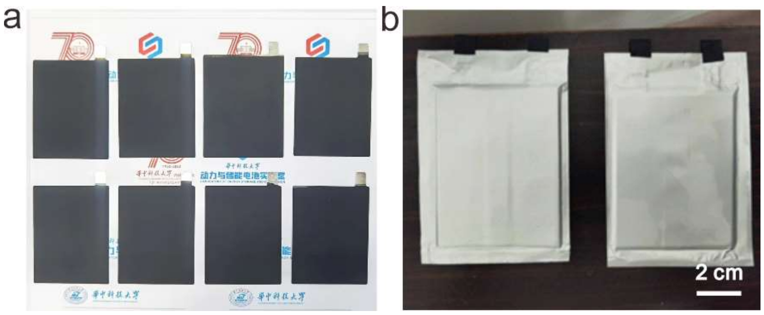

Supplementary Fig. 32 Digital photograph of (a) the large-scale preparation of SPAN@SPE and (b) the integrated SPAN|PTMG-HDI-BHDS|Li pouch cell.

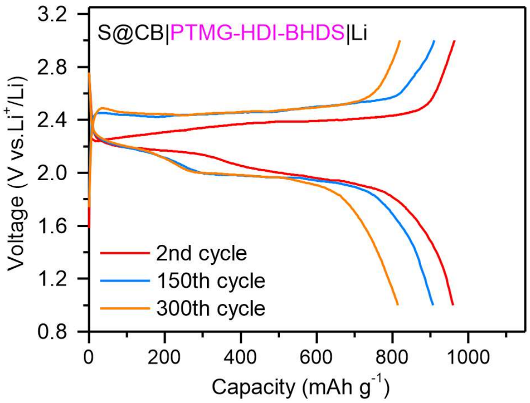

Supplementary Fig. 33 Charge/discharge curves of the S@CB|PTMG-HDI-BHDS|Li cell at 0.3 C.

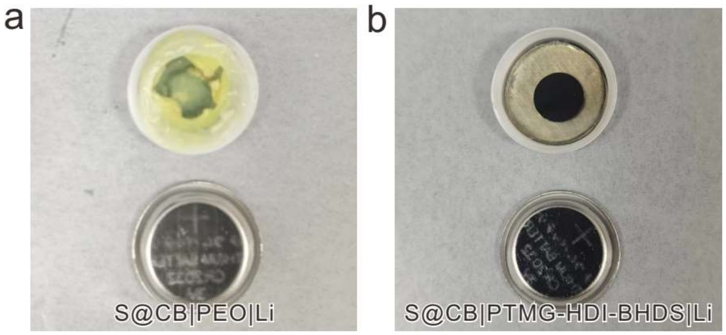

Supplementary Fig. 34 Digital photographs of the diffusion of polysulfides electrolytes after disassembled the (a) S@CB|PEO|Li and (b) S@CB|PTMG-HDI-BHDS|Li cells.

In particular, observing the diffusion of polysulfides in the liquid or gel electrolyte in H-type cell provides the most intuitive evidence for the study of shuttle effect in traditional Li-S batteries. But in this work, introducing polysulfide solution on both sides of the PTMG-HDI-BHDS/LiFSI in H-type cell will lead to significant swelling and destruction of the SPE, this will not be a true reflection of whether significant shuttle effects occur in solid-state electrolytes. To illustrate this problem, as shown in the Supplementary Fig. 34, the large amount of dissolved yellow polysulfides adhere to the surface of PEO/LiFSI after the cycling tests, however, there is no noticeable yellow color was observed in the PTMG-HDI-BHDS/LiFSI after disassembled the battery. Significantly lower concentration of ether-oxygen structure in PTMG-HDI-BHDS can effectively suppress the shuttling of polysulfides, agreeing well with the high capacity retention.

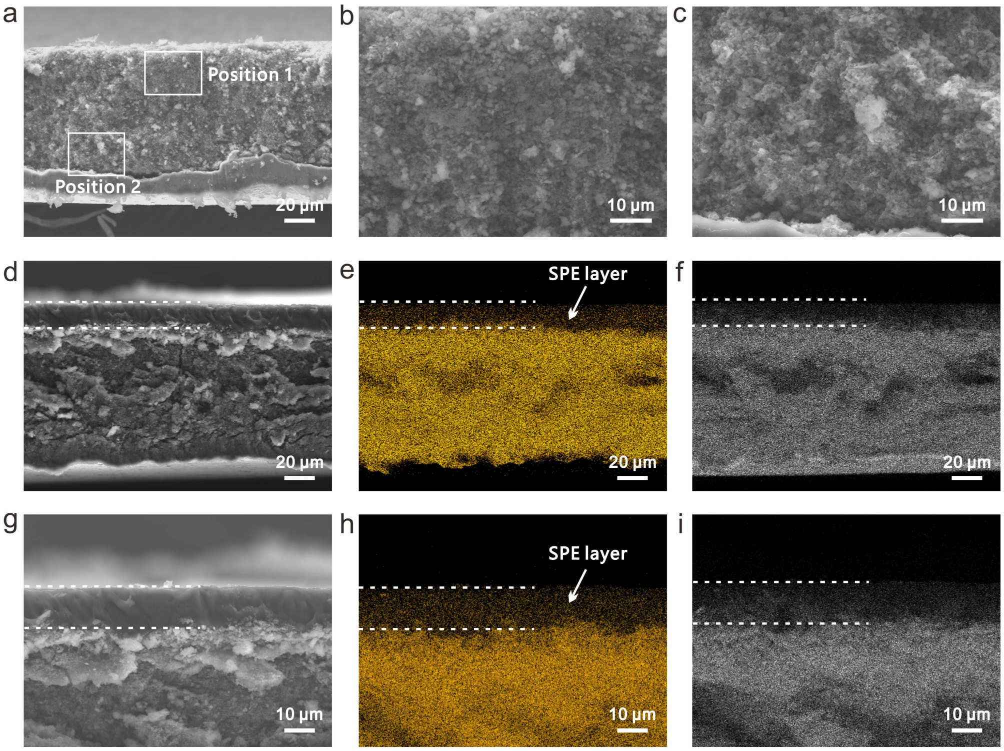

Supplementary Fig. 35 Morphology of integrated SPAN@SPE. (a-c) The cross-sectional SEM images of the thicker SPAN cathode. (d) The cross-sectional SEM image of integrated SPAN@SPE. (e, f) The corresponding S mapping and backscattered-electron of integrated SPAN@SPE. (g) The magnifying cross-sectional SEM image of integrated SPAN@SPE. (h, i) S mapping and backscattered-electron of integrated SPAN@SPE.

We had introduced PTMG-HDI-BHDS/LiFSI as a binder and Li^+ conductor into thick SPAN cathode to transport Li^+ (6.8 mg cm^{-2} with a thickness of ~80 μm). The dried cathode was rolled to minimize the porosity and increase electron conduction, so the porosity was not considered in the work (Supplementary Fig. 35a-c). The SPE solution was indeed difficult to penetrate into the rolled cathode, Li^+ pathways in thick SPAN cathode were provided by the designed SPE binder. The continuous electrode/electrolyte interface was formed without any cracks in the S mapping images (Supplementary Fig. 35e and 35h). It was worth noting that the SPE layer and the SPAN layer both contain light elements C, N, O, F, N and S, which were difficult to distinguish in the backscattered-electron SEM images (Supplementary Fig. 35f and 35i).

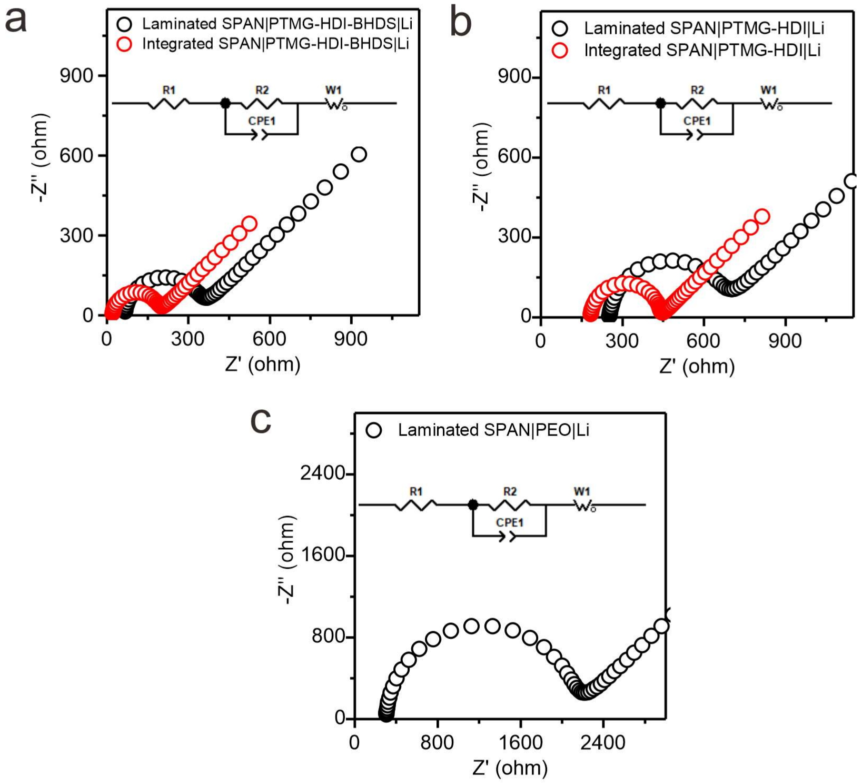

Supplementary Fig. 36 Impedance comparison of different battery assembly methods. EIS plots of the (a) SPAN|PTMG-HDI-BHDS|Li, (b) SPAN|PTMG-HDI|Li and (c) SPAN|PEO|Li with different fabrication strategies.

Systematic EIS studies with circuit models were performed on PEO/LiFSI, PTMG-HDI/LiFSI and PTMG-HDI-BHDS/LiFSI with different fabrication strategies. The impedance of these SPAN|SPEs|Li decreased overall in the order of PEO/LiFSI > PTMG-HDI/LiFSI > PTMG-HDI-BHDS/LiFSI, agreeing well with their ionic conductivities. It is worth noting that the dual-integrated strategy cannot be adopted with PEO/LiFSI because the significant side reaction between the solvent of acetonitrile and Li (PEO was dissolved in acetonitrile). For PTMG-HDI/LiFSI and PTMG-HDI-BHDS/LiFSI, as shown in the Supplementary Fig. 36, the dual-integrated strategy remarkably reduced the interfacial resistance between SPEs and electrodes compared to the laminated strategy. Overall, the ionic conductivity of the SPEs plays a crucial and decisive role in improving the electrochemical performance of the battery.

Supplementary Table 1. The cycling stabilities of the solid-state Li|SPEs|Li symmetric cells.

Electrolytes	Current density/capacity (mA cm^{-2}/mAh cm^{-2})	Cycling time (h)	Rate capability (mA cm^{-2})	References
PVFH-VEC-SN	0.2/0.2 (26 °C)	1500	1	Ref. 2
PVDF-hfp/SiO_2	0.2/0.2 (25 °C)	3000	0.5	Ref. 3
PAN/$Li_{1.4}Al_{0.4}Ti_{1.6}(PO_4)_3$/PEO-LiTFSI	0.2/0.2 (60 °C)	1000	0.2	Ref. 4
PEO/LLZTO-LiTFSI	0.2/0.1 (55 °C)	600	0.2	Ref. 5
PEO-LiFTFSI	0.1/0.2 (70 °C)	200	0.1	Ref. 6
PEGMEA-$Li_7La_3Zr_2O_{12}$-LiTFSI	0.1/0.1 (55 °C)	3200	0.1	Ref. 7
PEO/PMA	0.1/0.1 (65 °C)	336	0.1	Ref. 8
PEO/$Li_{3/8}Sr_{7/16}Ta_{3/4}Zr_{1/4}O_3$-LiTFSI	0.1/0.1 (45 °C)	700	0.6	Ref. 9
PE/PEGMEA	0.1/0.1 (60 °C)	1500	0.45	Ref. 10
PI/DBDPE/PEO-LiTFSI	0.1/0.1 (60 °C)	300	0.1	Ref. 11
PI/PEO-LiTFSI	0.1/0.1 (60 °C)	1000	0.1	Ref. 12
PEGDA-LiTFSI/LiBOB	0.05/0.05 (30 °C)	1300	0.05	Ref. 13
PE/PEO-LiTFSI	0.1/0.1 (60 °C)	1500	0.1	Ref. 14
**PTMG-HDI-BHDS-LiFSI**	**0.2/0.2 (30 °C)**	**5500**	**1**	**This work**

Supplementary Table 2. Comparison of cycling performance of solid-state Li-S batteries.

**Electrolytes**	**S cathode**	**S loading (mg cm^{-2})**	**Capacity after the cycling test (mAh g^{-1})**	**References**
PE/PEO-LiTFSI	S	1.0 (26 ^oC)	~625 mAh g_S^{-1} (30th cycle at 0.1 C)	Ref. 14
Poly(DOL)/PEG/SiO_2-LiTFSI	SPAN	0.5 (60 ^oC)	~500 mAh g_S^{-1} (100th cycle at 0.1 C)	Ref. 15
PEO/PVDF-LiTFSI	S	~1.0 (55 ^oC)	~650 mAh g_S^{-1} (60th cycle at 0.1 C)	Ref. 16
PEO/LGPS-LiTFSI	SPAN	~1.0 (60 ^oC)	~588 mAh g_S^{-1} (50th cycle at 0.1 C)	Ref. 17
PEO/CNT/GO-LiTFSI	S	0.5 (60 ^oC)	800 mAh g_S^{-1} (50th cycle at 0.2 C)	Ref. 18
PEO/LLZO-$LiClO_4$	S	0.6-0.9 (37 ^oC)	~800 mAh g_S^{-1} (200th cycle at 0.08 C)	Ref. 19
PEO-PIM-LiTFSI	S	1.0 (26 ^oC)	~720 mAh g_S^{-1} (100th cycle at 0.5 C)	Ref. 20
PEO-LiFTFSI	S	~1.0 (70 ^oC)	~750 mAh g_S^{-1} (60th cycle at 0.1 C)	Ref. 6
PEO-PAN-LiTFSI	S	~1.0 (70 ^oC)	~752 mAh g_S^{-1} (75th cycle at 0.1 C)	Ref. 21
PIN/SN-LiTFSI	S	~1.0 (70 ^oC)	~420 mAh g_S^{-1} (50th cycle at 1 C)	Ref. 22
LLZO/PEO-LiTFSI	S	0.41 (45 ^oC)	820 mAh g_S^{-1} (50th cycle at 0.073 C)	Ref. 23
**PTMG-HDI-BHDS-LiFSI**	**SPAN**	**2~2.2 (30 ^oC)**	**745 mAh g_{SPAN}^{-1} (700th cycle at 0.3 C)**	**This work**
	**S**	**2~2.2 (30 ^oC)**	**782 mAh g_S^{-1} (350th cycle at 0.3 C)**	

References:

1. Jiang C. et al. Regulating the solvation sheath of Li ions by using hydrogen bonds for highly stable lithium-metal anodes. *Angew. Chem. Int. Ed.* **133**, 10966-10974 (2021).
2. Wen, K. H. et al. Ion-dipole interaction regulation enables high-performance single-ion polymer conductors for solid-state batteries. *Adv. Mater.* **34**, 2202143 (2022).
3. Zhang, T. et al. A silica-reinforced composite electrolyte with greatly enhanced interfacial lithium-ion transfer kinetics for high-performance lithium metal batteries. *Adv. Mater.* **34**, 2205575 (2022).
4. Liang, J. Y. et al. Engineering janus interfaces of ceramic electrolyte via distinct functional polymers for stable high-voltage Li-metal batteries. *J. Am. Chem. Soc.* **141**, 9165-9169 (2019).
5. Huo, H. Y. et al. Rational design of hierarchical “ceramic-in-polymer” and “polymer-in-ceramic” electrolytes for dendrite-free solid-state batteries. *Adv. Energy Mater.* **9**, 1804004 (2019).
6. Eshetu, G. G. et al. Ultrahigh performance all solid-state lithium sulfur batteries: salt anion’s chemistry-induced anomalous synergistic effect. *J. Am. Chem. Soc.* **140**, 9921-9933 (2018).
7. Duan, H. et al. Dendrite-free Li-metal battery enabled by a thin asymmetric solid electrolyte with engineered layers. *J. Am. Chem. Soc.* **140**, 82-85 (2018).
8. Zhou, W. D. et al. Double-layer polymer electrolyte for high-voltage all-solid-state rechargeable batteries. *Adv. Mater.* **31**, 1805574 (2019).
9. Xu, H. H. et al. High-performance all-solid-state batteries enabled by salt bonding to perovskite in poly (ethylene oxide). *Proc. Natl Acad. Sci. USA* **116**, 18815-18821 (2019).
10. Wang, Z. Y., Shen, L., Deng, S. G., Cui, P. & Yao, X. Y. 10 μm-thick high-strength solid polymer electrolytes with excellent Interface compatibility for flexible all-solid-state lithium-metal batteries. *Adv. Mater.* **33**, 2100353 (2021).
11. Cui, Y. et al. A fireproof, lightweight, polymer-polymer solid-state electrolyte for safe lithium batteries. *Nano letters* **20**, 1686-1692 (2020).
12. Wan, J. Y. et al. Ultrathin, flexible, solid polymer composite electrolyte enabled with aligned nanoporous host for lithium batteries. *Nat. Nanotechnol.* **14**, 705-711 (2019).
13. Li, S. et al. A superionic conductive, electrochemically stable dual-salt polymer electrolyte. *Joule* **2**, 1838-1856 (2018).
14. Wu, J. Y. et al. Ultrathin, flexible polymer electrolyte for cost-effective fabrication of all-solid-state lithium metal batteries. *Adv. Energy Mater.* **9**, 1902767 (2019).

15. Utomo, N. W., Deng, Y., Zhao, Q., Liu, X. T. & Archer, L. A. Structure and evolution of quasi-solid-state hybrid electrolytes formed inside electrochemical cells. *Adv. Mater.* **34**, 2110333 (2022).
16. Fang, R. Y. et al. Reaction mechanism optimization of solid-state Li-S batteries with a PEO-based electrolyte. *Adv. Funct. Mater.* **31**, 2001812 (2021).
17. Li, M. R. et al. Solid-state lithium-sulfur battery enabled by Thio-LiSICON/polymer composite electrolyte and sulfurized polyacrylonitrile cathode. *Adv. Funct. Mater.* **30**, 1910123 (2020).
18. Liu, Y. et al. Mechanistic investigation of polymer-based all-solid-state lithium/sulfur battery. *Adv. Funct. Mater.* **31**, 2104863 (2021).
19. Tao, X. Y. et al. Solid-state lithium-sulfur batteries operated at 37 ^{o}C with composites of nanostructured $Li_7La_3Zr_2O_{12}$/carbon foam and polymer. *Nano letters* **17**, 2967-2972 (2017).
20. Ji, Y. C. et al. PIM-1 as a multifunctional framework to enable high-performance solid-state lithium-sulfur batteries. *Adv. Funct. Mater.* **31**, 2104830 (2021).
21. Sheng, J. Z. et al. Crosslinked nanofiber-reinforced solid-state electrolytes with polysulfide fixation effect towards high safety flexible lithium-sulfur batteries. *Adv. Funct. Mater.* **32**, 2203272 (2022).
22. Dong, D. R. et al. Polymer electrolyte glue: A universal interfacial modification strategy for all-solid-state Li batteries. *Nano letters*, **19**, 2343-2349 (2019).
23. Song, Y. X. et al. Direct tracking of the polysulfide shuttling and interfacial evolution in all-solid-state lithium-sulfur batteries: a degradation mechanism study. *Energy Environ. Sci.* **12**, 2496-2506 (2019).
